# A fast and accurate method to detect allelic genomic imbalances underlying mosaic rearrangements using SNP array data

**DOI:** 10.1186/1471-2105-12-166

**Published:** 2011-05-17

**Authors:** Juan R González, Benjamín Rodríguez-Santiago, Alejandro Cáceres, Roger Pique-Regi, Nathaniel Rothman, Stephen J Chanock, Lluís Armengol, Luis A Pérez-Jurado

**Affiliations:** 1 Center for Research in Environmental Epidemiology (CREAL), Doctor Aiguader 88, Barcelona 08003, Spain; 2 Institut Municipal d'Investigació Mèdica (IMIM), Doctor Aiguader 88, Barcelona 08003, Spain; 3 CIBER Epidemiología y Salud Pública (CIBERESP), Spain; 4 Dept. de Ciències Experimentals i de la Salut, UPF, Barcelona 08003, Spain; 5 CIBER de Enfermedades Raras, CIBERER, Spain; 6 Department of Human Genetics, University of Chicago, IL 60637, USA; 7 Division of Cancer Epidemiology and Genetics, National Cancer Institute, Bethesda, MD 20852-4907, USA; 8 Core Genotyping Facility, SAIC-Frederick, Frederick, MD 21702, USA; 9 Quantitative Genomic Medicine Laboratories, Ltd (qGenomics), Doctor Aiguader 88, Barcelona 08003, Spain

## Abstract

**Background:**

Mosaicism for copy number and copy neutral chromosomal rearrangements has been recently identified as a relatively common source of genetic variation in the normal population. However its prevalence is poorly defined since it has been only studied systematically in one large-scale study and by using non optimal *ad-hoc *SNP array data analysis tools, uncovering rather large alterations (> 1 Mb) and affecting a high proportion of cells. Here we propose a novel methodology, Mosaic Alteration Detection-MAD, by providing a software tool that is effective for capturing previously described alterations as wells as new variants that are smaller in size and/or affecting a low percentage of cells.

**Results:**

The developed method identified all previously known mosaic abnormalities reported in SNP array data obtained from controls, bladder cancer and HapMap individuals. In addition MAD tool was able to detect new mosaic variants not reported before that were smaller in size and with lower percentage of cells affected. The performance of the tool was analysed by studying simulated data for different scenarios. Our method showed high sensitivity and specificity for all assessed scenarios.

**Conclusions:**

The tool presented here has the ability to identify mosaic abnormalities with high sensitivity and specificity. Our results confirm the lack of sensitivity of former methods by identifying new mosaic variants not reported in previously utilised datasets. Our work suggests that the prevalence of mosaic alterations could be higher than initially thought. The use of appropriate SNP array data analysis methods would help in defining the human genome mosaic map.

## Background

Microarray platforms based on Single Nucleotide Polymorphisms (SNP arrays) are powerful tools in the research of genomic structural variation because they allow the integration of genotype and copy-number information. Researchers can simultaneously identify both copy number and copy number neutral changes, using the log2-ratio (LRR) intensity signal and the allele genotyping of the probes [1-6]. While SNP arrays have been effectively used in the study of copy number variation (CNV) and single nucleotide polymorphism (SNP) genotyping, only recently they have been utilised to identify the mosaic occurrence of copy number and copy neutral genomic abnormalities [7]. Genetic mosaicism is recognized as the presence of two or more different cell populations with different genotypes in one single individual, developed from a single fertilized egg. Such genetic abnormalities may result from a mutation during development that is propagated to only a subset of the adult cells. Somatic mosaicism for chromosomal rearrangements has been recently described on the basis of comparative analysis of differentiated human tissues from adult individuals [8] and divergence between identical twins [9]. Moreover it is well known that some mosaic abnormalities are involved in multiple developmental and tissue-specific disorders [10-16]. Despite of all these evidences, the frequency and extent of chromosomal mosaicism in adult normal population has been estimated only recently for the first time [17], and its real contribution to intra- and inter-individual genome variation is yet to be determined. For helping in that purpose, specialized algorithms and data analysis tools aimed at calling the mosaic occurrence of structural variation are badly needed.

Mosaic events can be captured by analysing SNP array data, specially from assessing multiple clusters of heterozygous alleles showing B allele frequency (BAF) and LRR values different from the those expected for regular heterozygous deletions, duplications or loss of heterozygosis events (See Additional File 1 - Figure S1 for examples of different types of mosaic rearrangements). Two recent studies have demonstrated that structural variants occurring in mosaicism are more frequent than expected, and thus they may play a relevant role in human diversity and disease susceptibility [7,17]. While both studies used Illumina SNP array data, only Rodríguez-Santiago et al. (2010) used tools for discovering occurrences in a systematic way. Their approach may, however, result in the underestimation of mosaic prevalence in two challenging situations: 1) small rearrangements and 2) rearrangements affecting a low percentage of cells. In addition, the used algorithm was computationally demanding (2 weeks to analyze about 2,000 individuals genotyped with Illumina HumanHap 1M), which constitutes a technical drawback in the analysis of high-density arrays of thousands of individuals.

Mosaicism detection can be cast as a segmentation problem [18]. In fact BAFsegmentation is a software developed for the identification of mosaicism in cancer cells based on the circular binary segmentation (CSB) algorithm [19]. The disadvantage of this procedure is the lack of a method to clearly control the false discovery rate (FDR) [20].

As an alternative segmentation method, the genome alteration detection analysis (GADA) can also identify allelic imbalances by using BAF values provided by SNP arrays [21]. This value is the fraction of the total signal due to a specific allele and it is the suited value to study allelic imbalances underlying mosaicism for genomic rearrangements. Compared to circular binary segmentation (CBS) [19], GADA has similar accuracy, and is several orders of magnitude faster. Recently, GADA segmentation was applied to CNV calling by using LRR values from SNP array data in very large data sets with high efficiency and accuracy [6,22]. To overcome the specific difficulties in the identification of mosaic events from SNP arrays, we have developed Mosaic Alteration Detection-MAD method which includes both statistic (including FDR control) and bioinformatic tools to specifically analyse BAF values from SNP array data. The software presented here improves SNP array data analysis allowing the capture of mosaic copy number (deletions, duplications, aneuploidies) and copy neutral changes (uniparental disomies, UPD, namely the occurrence of two copies of a particular chromosome from the same parent), as well as regions of homozygosity due to identity-by-descent. The developed method was used to analyse Illumina HumanHap 1M SNP array data obtained from control, bladder cancer and HapMap individuals. We also compared its performance (sensitivity and specificity) with BAFsegmentation [18] under several simulated challenging scenarios like small altered regions, low percentage of mosaic cells and poor array quality. Finally, our tool was applied to SNP array data previously utilised for detecting mosaic abnormalities [17,18]. While all published findings were successfully identified, additional mosaic events were detected by MAD and experimentally validated afterwards. Our results suggest that improved methods can accurately capture mosaic chromosomal rearrangements using SNP array data and that their prevalence is higher than initially thought.

## Results and Discussion

### Method development

**Figure 1:**
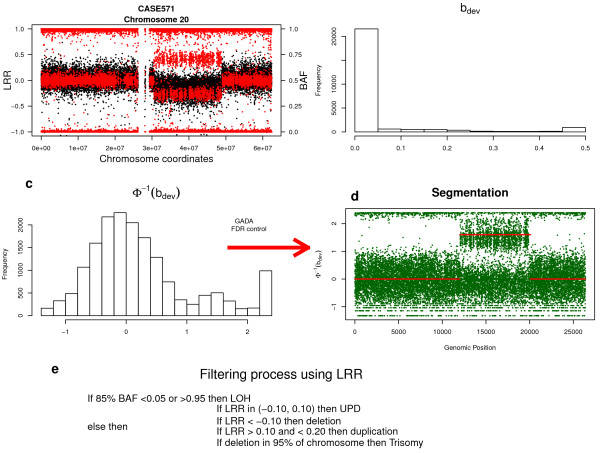
**Algorithm process to detect genomic imbalances using SNP array data**. (a) B allele frequency (BAF, red dots) from heterozygous clusters different to non altered probes (BAF 0.5) can be used to iddentify genomic imbalances. (b) b-deviation, *b*_dev_, is computed to detect altered regions. (c) Probit transformation, Φ^-1^(*b*_dev_), is used to achieve normallity before applying a segmentation algorithm. (d) Segmentation algorithm detects altered regions. False discovery rate (FDR) can be controlled in this step. (e) After calling segments by using BAF values, LRR allows to classify altered regions in different kind of mosaic structural variations.

Figure 1 contains a scheme showing the overall performance of the MAD algorithm, using CASE571 carrying a mosaic deletion in chromosome 20 from Mb 31 to 48, as an example. The distribution of LRR and BAF values for the individual can be visualised in Figure 1a. Two clusters of BAF values (in red) are clearly visible between MB 31 to 48 that would be absent in a standard heterozygous deletion. Therefore, the abnormality could be detected by the deviation of the BAF signal from the expected values typical for non-altered homozygous (1 or 0) or non-altered heterozygous probes (0.5). The difference between observed and expected BAF is denoted as b-deviation. Altered regions can be called by detecting segments with b-deviation values different from zero using algorithms such as GADA and CBS. Segmentation methods usually assume that the data are normally distributed. However this is not the case for b-deviation as Figure 1b demonstrates. The further variable Φ^-1 ^(Figure 1c), which is a probit transformation on b-deviation having a normal distribution, can be applied to perform the segmentation procedure (Figure 1d) controlling the FDR parameter. Once the segments have been called by assessing BAF values as described above, the average LRR of the called segment is calculated to help in determining whether the abnormality is affecting the copy number (Figure 1e).

### New mosaic rearrangements from previously utilised SNP array datasets

#### HapMap individuals

**Figure 2:**
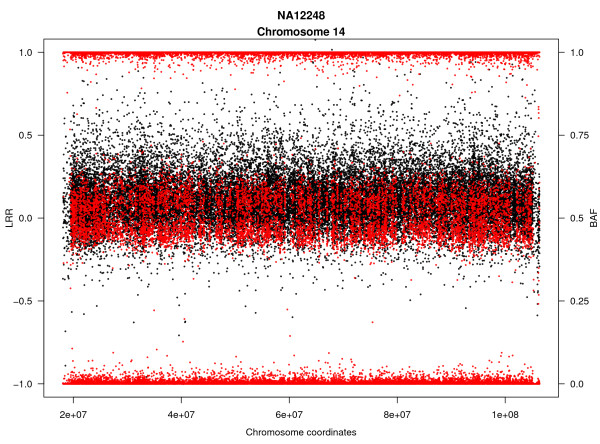
**Example of mosaic rearrangement detected in HapMap individuals**. The plot shows a trisomy in chromosome 14 for the individual NA12248 detected using MAD (not detected using BAFsegmentation). The same alteration was also identified when analysing a replicated experiment of the same HapMap individual. Red dots represent B-allele frequency (BAF), while black dots show log2ratio (LRR) values.

To check how the algorithm works with real data MAD tool was applied to data obtained from lymphoblastic cell line DNA of 125 individuals from HapMap population (60 CEU, 60 YRI and 5 replicates) hybridized with Illumina HumanHap 1M SNP array, available at public repositories http://www.hapmap.org. For MAD, parameters were set to *T *= 8, *a *= 0.8 (see Method section for a definition of these two parameters and Additional File 2 for a table including the recommended settings for being used in a preliminary scan) and the minimum probe length for detection at MinSegLen = 300, meaning the algorithm is able to detect altered regions > 0.9 Mb for 1 Mb arrays. Furthermore, with *a *= 0.8 finding few number of segments for each individual is expected. With these parameters, the FDR was < 0.0001. The tool identified 9 mosaic rearrangements in 8 individuals (Table 1). Mosaic uniparental disomy was observed for chromosome 2 in one individual (NA18855). Mosaic duplications were detected in three individuals affecting chromosome 2 (NA10857), chromosome 19 (NA11882) and chromosome 8 (NA18972). Gains of the entire chromosome compatible with mosaic trisomies were observed in 4 individuals (NA11236, NA12248, NA12875 and NA19193) involving chromosomes 15, 9 and 14, 2 and 12, respectively and different percentage of mosaic cells (see Additional File 3). In addition the performance of MAD was compared to BAFsegmentation using default parameters in the HapMap individuals analysed. Compared to MAD, BAFsegmentation algorithm was unable to detect 2 alterations, trisomies with very low proportion of affected cells (see Figure 2 for one case). The obtained results are consistent with the simulation analysis performed in the present work where BAFsegmentation showed less sensitivity for low mosaic cell proportions (see section below).

**Table 1:** Summary of mosaic structural variants identified in HapMap individuals by using MAD tool.

Sample	BAFsegmentation?	Chr	Event	Start	End	Size (Mb)
NA10857	Yes	2	Duplication	59,505,990	60,622,482	1.1
NA11882	Yes	19	Duplication	57,313,456	63,802,440	6.7
NA12236	Yes	15	Trisomy	pter	qter	
NA12248	No	14	Trisomy	pter	qter	
NA12248	No	9	Trisomy	pter	qter	
NA12875	Yes	2	Trisomy	pter	qter	
NA18855	Yes	2	UPD	pter	94,755,295	94,7
NA18972	Yes	8	Duplication	4,669,277	5,914,260	1.2
NA19193	Yes	12	Trisomy	pter	qter	

The second column indicates whether BAFsegmentation algorithms also found the alteration.

#### Control and bladder cancer individuals

The tool was also used to re-analyse previously defined and validated mosaic rearrangements reported elsewhere [17]. A selected subset of 31 mosaic rearrangements among 58 samples was initially studied. Parameters were set to *T *= 8, *a *= 0.8 and MinSegLen = 1500 that are predicted to have a FDR of 0.0001.

Using these settings the tool was able to detect all previously defined mosaic rearrangements but one (30/31). Remarkably, the algorithm did not provide any false-positive calls. The false-negative result was a small deletion on chromosome 20 only comprised by 248 probes (that is not possible to be detected with MinSegLen = 1500). Nonetheless, this alteration was detected by changing MinSegLen to 200, and no other abnormality was found in this sample by using this setting. Overall, the predicted FDR using MinSegLen = 200 is 0.001. The MinSegLen is a parameter that can be adjusted depending on probe coverage of the array, and it can be reduced for hihg-density arrays such as Illumina HumanHap 1M. The complete dataset of 1991 samples was studied afterwards [17]. We set MinSegLen = 100 and *T *= 8, to increase power detection at the expense of higher FDR (0.05). The algorithm detected 7 new segments not identified either with previous tools or with BAFsegmentation (Table 1 and Additional File 1 - Figures S5 to S11). These abnormalities were smaller in size than those previously reported, with the minimum size of ~ 500 Kb (See Additional File 1 - Figure S5 for an example) which represents an improvement in the detection of smaller events. We experimentally validated the mosaic abnormalities in all 6 available DNA samples by multiple ligase-dependent probe amplification (MLPA) (Table 2 and Additional File 1 - Figures S12 to S17). The algorithm also detected 98 duplication segments ranging in size from ~ 0.2 to ~ 4 Mb with average LRR < 0.15 suggestive of possibly being mosaics (Additional File 4).

**Table 2:** Eight new mosaic events identified in SNP array data from individuals previously analysed in Rodriguez-Santiago et al. (2010).

Sample	Chr	Event	Start	End	Size (Mb)	MLPA validation^1^
CONTROL870	2	Deletion	25,056,172	25,570,101	513,929	Yes
CONTROL1210	2	Deletion	25,312,050	26,013,444	701,394	Yes
CASE623	4	Duplication	158,590,621	159,723,224	1,132,603	Yes
CASE741	6	Duplication	162,534,963	163,052,519	517,556	NA
CASE526	7	Deletion	138,459,817	139,610,819	1,151,002	Yes
CASE508	11	Duplication	122,663,431	123,143,910	488,481	Yes
CONTROL413	15	Duplication	48,349,855	48,838,336	517,556	Yes

The new abnormalities observed were smaller than those previously reported. Figures are available in the Additional File 6 - Figures S5 to S11.

^1 ^Additional File 5 (Section 4) shows the MLPA electropherograms and control comparisons.

### Simulation Studies

Several simulation studies were run to further assess the performance of MAD and BAFsegmentation analysis tools. Specifically, eight scenarios were considered depending on i) the percentage of affected cells with the altered region: 10% and 20%; ii) the length of the alteration: small and large; and iii) the quality of data: good and noisy. The BAF value was simulated for 20,000 SNPs using a log-normal distribution with mean 0 for AA homozygous and mean 1 for BB homozygous probes. A normal distribution with mean 0.5 was used to generate heterozygous markers. Different quality data was generated varying the variance of these distributions (0.03 for good quality and 0.1 for noisy). The percentage of cells with an abnormal region was simulated by changing the mean value for heterozygous probes (0.55 and 0.60 for 10% and 20% of affected cells, respectively). Finally, abnormal large regions were generated by modifying 10,000 markers, while small aberrant regions contained 1,000 probes. These simulations allowed the calculation of: 1) the ROC curve (True-positive Vs False positive rates); 2) the FDR; and 3) differences in sensitivities of MAD and BAFsegmentation. The results were based on 1,000 simulations.

#### True-positive (TPR) and False-positive (FPR) rates

**Figure 3:**
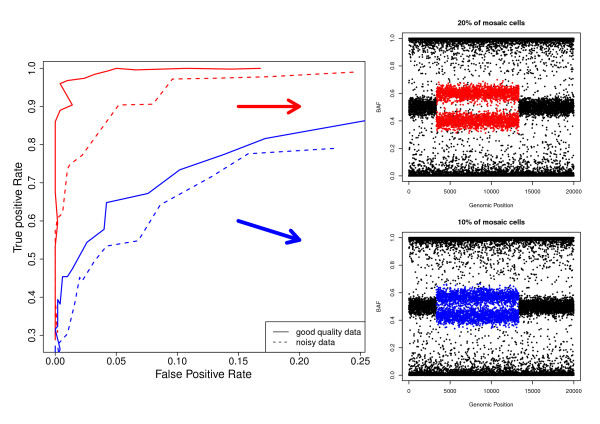
**ROC curve obtained from simulated data**. Four scenarios were considered depending on i) the percentage of mosaic cells in the altered region: 10% (blue lines) and 20% (red lines) and ii) the quality of data: good (solid lines) and noisy (dashed lines). Each line gives the True-positive rate for a given False-Positive rate level. This example corresponds to a case with moderate/large altered region.

The performance of MAD algorithm was examined under changes in the parameters, *a *∈ {0.2, 0.8} and *T *∈ {2.5, 3, 3.5, 4, 4.5, 5}, that control its sensitivity and specificity. Figure 3 shows the ROC curve (e.g. TPR vs FPR) for different simulated scenarios and for the case of having moderate to large altered regions. As expected, the TPR improves when both quality data and percentage of affected cells increase. The performance was almost perfect (e.g. area under the ROC curve is near 1) when quality data was good and the degree of mosaicism was at least 20% (continuous red line). Similar results were obtained when analyzing simulated segments with less density (1,000 abnormal SNPs - Additional File 1 - Figure S4).

#### False discovery rate (FDR)

**Figure 4:**
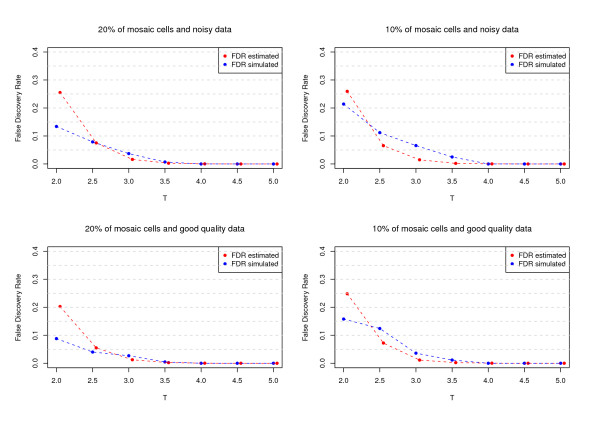
**False discovery rate (FDR) validation**. Each figure compares the simulated FDR vs. the estimated FDR using the approach given in the Methods Section for different scenarios. The percentage of mosaic cells in the altered region as well as the quality of the data were changed.

As a significant feature MAD incorporates a general method to control FDR. This method was validated by using simulated datasets and comparing estimated with expected FDR values. Figure 4 shows the FDR comparison for different scenarios under *a *= 0.8. A good agreement between simulated and estimated FDR in all situations was observed: the FDR decreased when either *T *, the percentage of affected cells or data quality increased. Similar results were obtained for *a *= 0.2, where the FDR is larger than the previous case, as expected.

#### Comparison with BAFsegmentation

The performance of MAD and BAFsegmentation tools was studied by simulating low cell proportion of mosaic alterations. A single segment of 1000 probes within a DNA chunk of 20-Kb in length was simulated 200 times for different proportion of mosaic cells (0, 0.01, ... 0.15). Parameters of both algorithms were fixed such as optimum specificity was achieved for both methods across the whole range. In other words, the algorithms were adjusted for not finding any segment which was completely disjointed from the one simulated. In particular, this was satisfied with *a *= 0.5, *T *= 2 and MinSegLen = 900 for MAD, and default values for BAFsegmentation. The comparison was assessed using the sensitivity of each method by measuring the proportion of identified segments covering at least 50% of the simulated segment.

**Figure 5:**
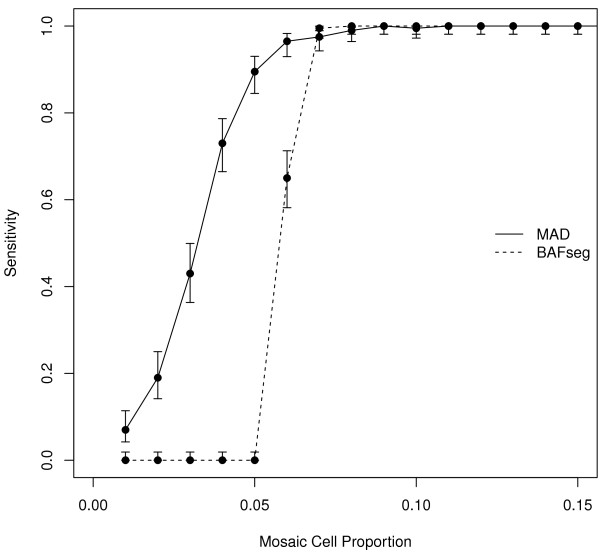
**Sensitivity as a function of mosaic cell proportion**. Low proportion of cells affected with the abnormality reduces the sensitivity to identify a 1 kB mosaic alteration, in a 20 kB region of 200 simulated individuals. Overall MAD showed a better performance when compared to BAFsegmentation.

Overall MAD showed a better performance when compared to BAFsegmentation as can be seen in Figure 5. BAFsegmentation achieved good sensitivity in the range of mosaic cell proportions > 0.07, and null sensitivity for values < 0.05. On the other hand, despite the lower sensitivity of MAD in the range (0.07, 0.15), there is an important amount on sensitivity captured in low values (0.02, 0.05) and a high sensitivity (0.98) at 0.15. The overall performance of both methods can be compared from the areas under each curve. In the case of the MAD curve the estimation of this area, normalized by the area of the perfect sensitivity curve (*y *= 1), is 0.109/0.15 = 0.73; whereas for BAFsegmentation this area is smaller (0.63). Therefore, under this scenario, MAD showed better sensitivity over the whole range of mosaic cell proportions. In addition, the computational time for analyzing the 58 samples described in previous sections was 3 min 15 sec when using MAD, while BAFsegmentation needed 42 min 50 sec.

## Conclusions

The accurate and appropriate analysis of SNP array data of genomic DNA from multiple cells allows for the identification of genomic changes occurring in mosaicism and subsequently for the estimation of the affected cell proportion. The assessment of this increasingly recognised type of genetic variation is relevant to define its impact over human diversity and clinical phenotypes. In this study, we have implemented the so called MAD tool to detect mosaic events from SNP arrays using the BAF value as a powerful parameter to detect the allelic imbalances that underlie mosaic alterations. Our method was successful in finding previously defined mosaic chromosomal alterations, and able to detect additional events in the same data set [17,18], which suggests a higher sensitivity for MAD. Remarkably, the tool was able to find mosaic rearrangements of smaller size (~ 500 Kb) and events affecting a lower proportion of cells, uncalled when using other algorithms.

The easy manipulation of the parameters *a *and *T *offers flexibility to the optimization of MAD for a wide range of circumstances. In contrast, a greater optimization of BAFsegmentation has yet to be developed for truly assessing the performace of the method, especially for small and low proportion of cells affected Another significant advantage of MAD is that the FDR can be directly handled by performing setting changes in its parameters. High sensitivity and low FDR are essential in assessing the prevalence of mosaic events in the available datasets. We have shown how to estimate the FDR from the segmentation output and, in our simulation studies, how close is such estimate is very close to the expected one. However, it is important to note that FDR also depends on the window for minimum probe length (MinSegLen). One possible limitation of our method is the increase in the number of false positives when reducing the (MinSegLen) for analysing arrays with lower probe coverage.

An unexpectedly increased number of duplications with respect to deletions were called by MAD. Most of these calls were not treated as mosaic because their features (size, LRR, Bdev, ...) or their plots did not suggest mosaic occurrence (see Additional File 4). While the distinction between constitutional and mosaic events is quite evident for deletions and UPDs, due to the finding of complete loss of heterozygosity only in non-mosaic rearrangements, in the case of copy number gains (duplications, trisomies) it is not so straightforward because the presence of heterozygous probes is expected. Due to the technical limitations in array platforms the change in copy number from 2 to 3 or an intermediate number could be in the same sensitivity range thus providing LRR and BAF array values similar for both normal gain dosage changes (2 to 3 i.e.) and mosaic changes occurring in a subset of cells. A comparison between MAD analysis and CNV calling process using PennCNV and filtering procedures revealed that 30 MAD duplications were absent in CNV dataset when a statistic filter was applied [23,24]. The 98 duplications showing any overlap in both datasets were 59% identical on average (range: 3% - 100%) (see Additional File 4). These results may suggest on one hand that MAD software seems to be more effective in calling duplications than typical CNV calling procedures and, in the other hand, that some duplication calls observed using MAD may be non mosaic alterations.

Reanalysis of the previously assessed SNP array data in [17] demonstrates that there are hidden genomic mosaic events that cannot be detected by using *ad-hoc *routines. The fact that such genomic allelic imbalances can be smaller and present in a lower proportion of cells, emphasizes the need of improving and using more powerful analytical methods. However, an open question regarding the detection limit remains partially dependent on the array resolution given that no gold standard exists to define the false negative rate for most rearrangements (other than FISH for large segmental or complete aneuplodies). While we have shown that MAD can successfully detect small and very low mosaicism degree events and that the prevalence of mosaicism can still be higher, further improvements are needed in the analysis and experimental techniques. Mosaicism is an unexpected source of genetic variation that is still underexplored. The study of existing and future SNP array datasets, as well as the application of similar algorithms for allelic imbalance detection to next-generation sequencing data, will provide new clues about the impact of such genetic variation over phenotypic differences in the common population as well as its influence on disease.

## Methods

### Analysis methods

#### Data

The simulated dataset was generated by varying i) the percentage of cells affected by a deletion type CNV (varying shift in B allele frequency to be 10% or 20%); ii) the length of the rearrangement, from large (half chromosome) to small (5% of the chromosome); and iii) the quality of the hybridization BAF signal data, that were defined as good (Additional File 1 - Figure S2) or noisy experiments (Additional File 1 - Figure S3).

The Illumina data set from HapMap individuals comprised 120 individuals and 5 replicates and were retrieved from http://www.illumina.com/. The data includes 60 CEPH (CEU) and 60 Yoruban (YRI) unrelated samples. The Illumina 1M HumanHap SNP array integrates about 1.0 million probes. Data were normalized by using Illumina BeadStudio software.

A subset of Illumina 1M HumanHap SNP array data obtanined from 58 individuals who participated in the Spanish Bladder Cancer study [25] kindly provided by the NCI Core Genotyping Facility was also analysed. These data have been already analyzed for mosaic events using different *ad-hoc *tools and manual curation [17]. All reported mosaic rearrangements were validated by additional molecular techniques such as multiplex ligation probe-dependent amplification (MLPA) and microsatellite marker analysis and/or FISH [17].

#### Algorithm

Allelic genomic imbalances (different proportions of the two homologous chromosome regions) can be detected from the BAF signal data (Figure 1). The transformed b-deviation value was calculated for each probe to detect genomic imbalances. The b-deviation of a probe, *b*_dev_, is defined as the deviation from the expected BAF given the genotype,(1)

Genomic imbalanced regions are captured by selecting SNPs with *b*_dev _different from 0. This is a segmentation problem that can be tackled with methods already available, most of which assume normality in the signal. Thus, the probit transformation, Φ^-1 ^, was used(2)

where erf denotes the error function (Figure 1C). Segmentation is then performed on regions with Φ^-1 ^≠ 0 using GADA which detects altered segments in two steps. The first step is a Sparse Bayesian Learning process (SBL) that generates a list of candidate breakpoints and segment means while trying to strike an optimal balance between model fit and model sparseness (the number of breakpoints). The SBL step is driven by two prior parameters *a *and *b *and they are directly controlled by the user. Typically *b *is set to zero as an uninformative prior, so sparseness is solely controlled by *a*, taking values between 0.2 and 0.8. By increasing *a *the algorithm has higher sensitivity but also higher FDR, which can be further adjusted in the second step. The significance of each called breakpoint segment is assessed with a *t*-statistic computed with the parameter estimatation provided by the SBL step. This statistic is a function of the segment mean and variance. The second step is then a backward elimination (BE) process which removes breakpoints with a level of significance (t statistic) lower than the user-defined threshold, [21] showed that, under the null hypothesis, if a segment is copy normal, the t-statistic distribution is normal across the segments. The ranking of breakpoints with the adjustment of T is obtained with very low computational cost and can be used to control the FDR.

After calling segments by assessing BAF values the average LRR is used to carry out a preliminary classification of the called segment into different types of mosaic rearrangements: UPD was considered for LRR ∈ {-0.10, 0.10}, a deletion when LRR < -.10, a duplication for LRR > 0.10 and a Trisomy if a deletion was observed in more than 95% of the chromosome. These values should not be absolute but relative to the average LRR of the entire diploid genome (exluding X and Y chromosomes) and the rest of the chromosome data.

#### False Discovery Rate estimation

Recommendations on how to choose *T *and *a *parameters to control both sensitivity and FDR, based on simulations and specific data (i.e., Affymetrix 500K, Illumina 550) have been given elsewhere [21]. Here, we propose a general method to control the FDR independent of reference arrays and resolution, derived from a previously reported one [26].

The hypothesis of no alteration for the *i*-th segment, *S_i_*, can be stated as(3)

where *μ_i _*denotes the mean of Φ^-1^(*b_dev _*) across all *n_i _*probes in *S_i_*. For each *S_i _*, GADA provides a statistic, *t_i _*. Under the null hyphotesis the distribution of *t_i _*is *N *(0, 1) for a large number of probes, whereas for a small number of probes it follows a *t*-Student distribution, *t_ν _*, with *ν *= *n_i _*- 1 degrees of freedom. If *p_k _*= *P *(*t *> |*t_i_*|) and *t_i _*are independent from each other, for a given *T *, a conservative estimator of the genome-wide FDR is(4)

where *N *is the total number of probes in the array.

#### Software

MAD is included in the R-GADA software [27] that is available at

http://www.creal.cat/jrgonzalez/software.htm. A detailed tutorial describing how to analyze SNP array data is available in the Additional File 5.

### Experimental validation

DNA samples were obtained from 6 out of 7 individuals showing mosaic abnormalities in Table 2. Validation of algorithm findings was carried out by using multiplex ligation-dependent probe amplification (MLPA) technique [28]. The MLPA reactions were carried out essentially as described previously [28] with slight modifications [29]. We used both visual examination of the electropherograms and the relative peak height (RPH) method recommended by MRC-Holland for data analysis [30]. The complete list and details of used MLPA probes is shown in Additional File 6.

## Competing interests

The authors declare no conflict of interest, excepting LA and LAPJ, executive director and member of the scientific advisory board of the qGenomics company, respectively.

## Authors' contributions

JRG, BR-S, LA and LAP-J conceived the idea of using B allele frequency value and a segmentation algorithm to detect mosaic alterarions from SNP array data. JRG and BR-S analyzed real data, and co-wrote the manuscript draft. BR-S and LAP-J interpreted and further analyzed real data results. JRG and RP-R implemented MAD algorithm and FDR procedure. JRG and AC designed, performed and interpreted simulation studies. NR, SJC and LAP-J supervised the project and participated in producing part of the array data analysed in the manuscript. All authors read and approved the final manuscript.

## Supplementary Material

Additional file 1 **File including figures for examples of simulated data sets, some simulation results and new mosaic abnormalities detected using MAD in SNP arrary data previously analyzed with ad-hoc tools (Rodriguez-Santiago et al., 2010)**. Click here for file

Additional file 2 **Recommended parameters for a preliminary scan using different Illumina platforms**. Click here for file

Additional file 3 **Comparison between MAD findings and chromosomal abnormalities previously described in HapMap individuals also analysed in Redon et al., Nature, 2006**. Click here for file

Additional file 4 **Additional analysis using PennCNV to discard mosaic detected with MAD with consitutional duplications**. Click here for file

Additional file 5 **User's guide of an R package that implements MAD algorithm including some real data examples**. Click here for file

Additional file 6 **List and details of used MLPA probes for validating new mosaic rearrangements**. Click here for file
