# Supplementary material for: A fast and accurate method to detect allelic genomic imbalances underlying mosaic rearrangements using SNP array data
Source: BMC Bioinformatics. 2011 May 17;12:166. doi: 10.1186/1471-2105-12-166 (PMC3118168; doi:10.1186/1471-2105-12-166)
Supplement: Additional file 1 — File including figures for examples of simulated data sets, some simulation results and new mosaic abnormalities detected using MAD in SNP arrary data previously analyzed with ad-hoc tools (Rodriguez-Santiago et al., 2010). [file 1471-2105-12-166-S1.PDF]

Supplementary Material: A fast and accurate method to detect allelic genomic imbalances underlying mosaic rearrangements using SNP array data

Juan R González, Benja Rodríguez-Santiago, Alejandro Cáceres, Roger Pique-Regi, Nathaniel Rothman, Stephen J Chanock, Lluís Armengol, Luis Pérez-Jurado

1 Examples of different types of mosaic rearrangements

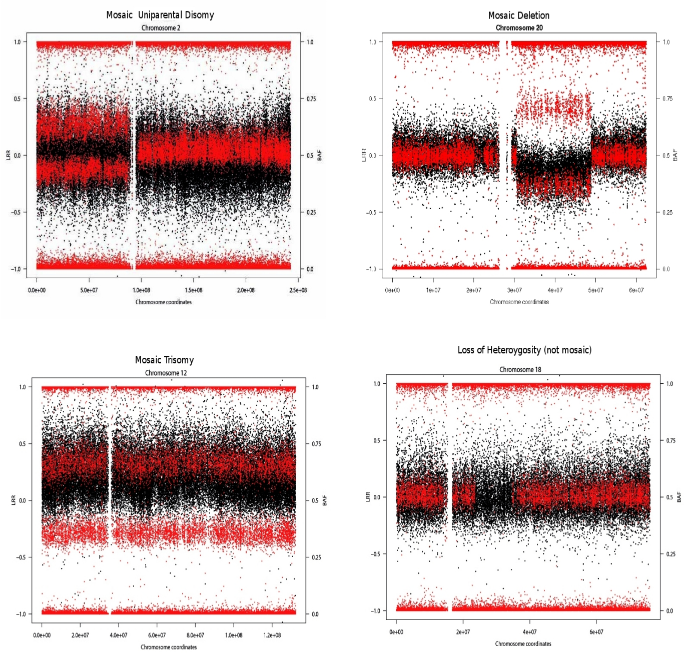

Figure 1: The plots show the LogR ratio (black dots, scale on the left side) and BAF (red dots, scale on the right side) values along the entire chromosome carrying the rearrangements in selected samples. We illustrate: 1) mosaic uniparental disomy (UPD) in distal 2p characterized by unchanged LogR and abnormal heterozygous BAF. 2) large mosaic deletion in chromosome arm 20q showing decreased LogR and abnormal heterozygous BAF without complete loss of heterozygosity. 3) Mosaic trisomy 12 with a pattern similar to that of duplications along the entire chromosome and 4) Copy neutral change, loss of heterozygosity reflected by an absence of heterozygous probes, unchanged LRR (LRR 0). This is a not mosaic abnormality.

2 Examples of simulated data sets

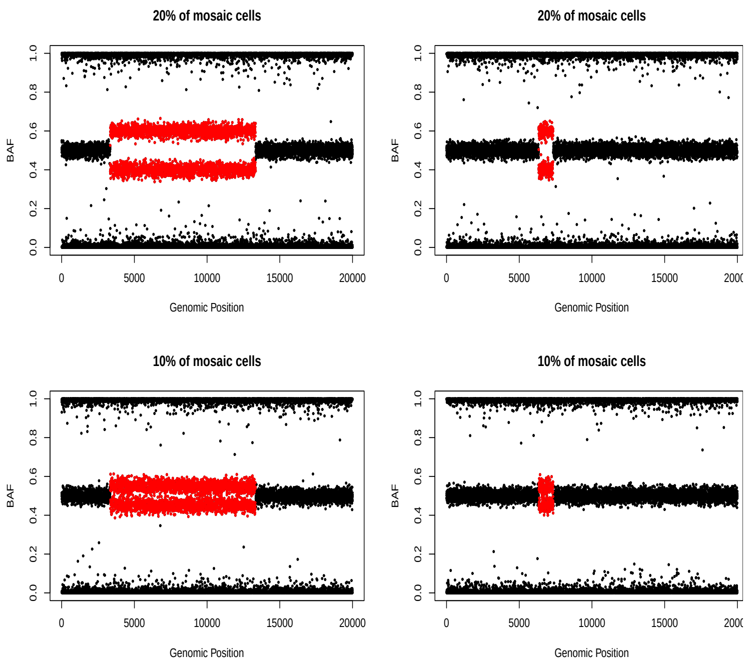

Figure 2: Example of simulated data set for the case of having good quality of the hybridization BAF signal data

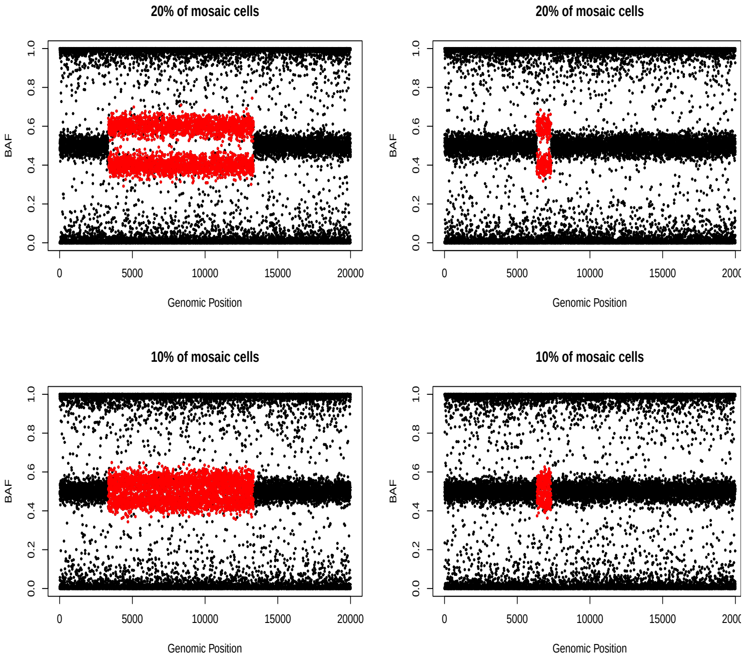

Figure 3: Example of simulated data set for the case of having bad quality of the hybridization BAF signal data

3 Simulation studies

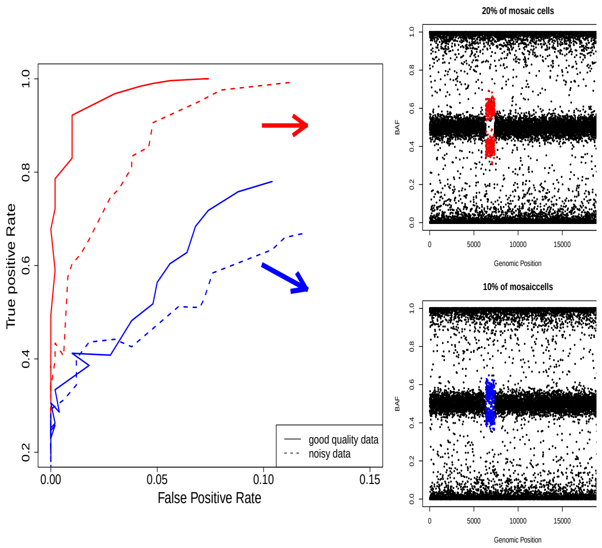

Figure 4: ROC curve in a simulated data. Four scenarios are considered depending on i) the percentage of mosaic cells in the altered region: 10% (blue lines) and 20% (red lines) and ii) the quality of data: good (solid lines) and noisy (dashed lines). Each line gives the True-positive rate for a given False-Positive rate level. This example corresponds to a case with small altered region

4 New mosaic abnormalities detected using MAD in SNP array data previously analyzed with ad-hoc tools (Rodriguez-Santiago et al., 2010)

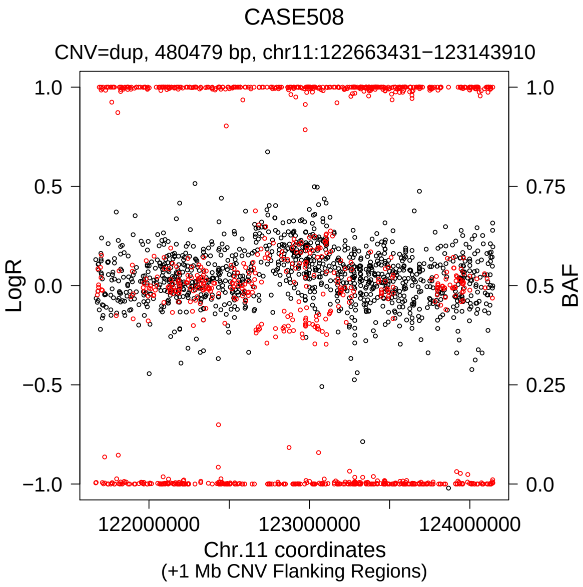

Figure 5: New mosaic rearrangement found after re-analyzing SNP array data described elsewhere (Rodriguez-Santiago et al. 2010). The plot shows a chromosome 11 duplication in sample CASE508 by using MAD. This was not detected using ad-hoc tools. Red dots represent B-allele frequency (BAF), while black dots show log2ratio (logR) values.

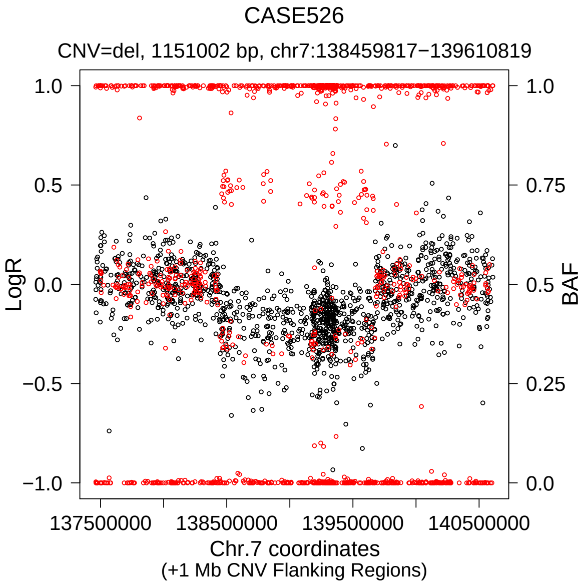

Figure 6: New mosaic rearrangement found after re-analyzing SNP array data described elsewhere (Rodriguez-Santiago et al. 2010). The plot shows a chromosome 7 deletion in sample CASE526 by using MAD. This was not detected using ad-hoc tools. Red dots represent B-allele frequency (BAF), while black dots show log2ratio (logR) values.

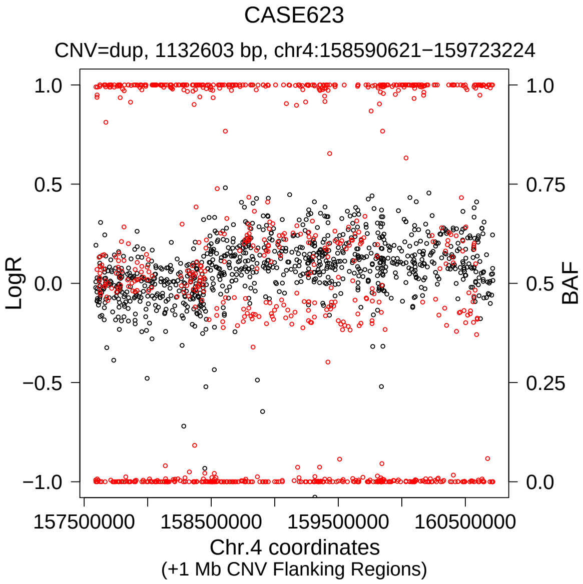

Figure 7: New mosaic rearrangement found after re-analyzing SNP array data described elsewhere (Rodriguez-Santiago et al. 2010). The plot shows a chromosome 4 duplication in sample CASE623 by using MAD. This was not detected using ad-hoc tools. Red dots represent B-allele frequency (BAF), while black dots show log2ratio (logR) values.

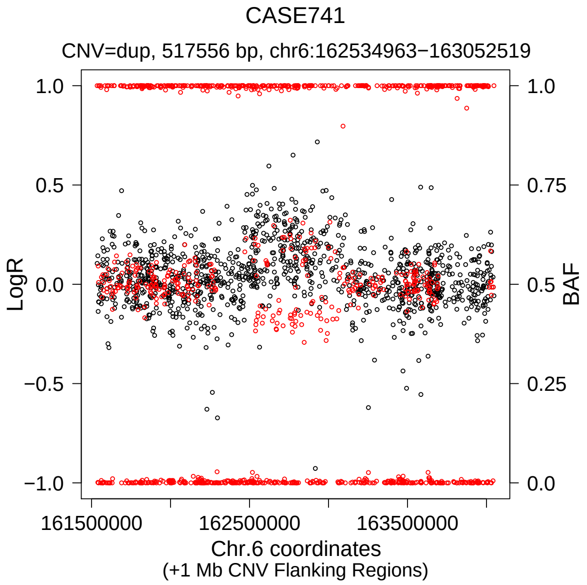

Figure 8: New mosaic rearrangement found after re-analyzing SNP arrary data described elsewhere (Rodriguez-Santiago et al. 2010). The plot shows a chromosome 6 duplication in sample CASE741 by using MAD. This was not detected using ad-hoc tools. Red dots represent B-allele frequency (BAF), while black dots show log2ratio (logR) values.

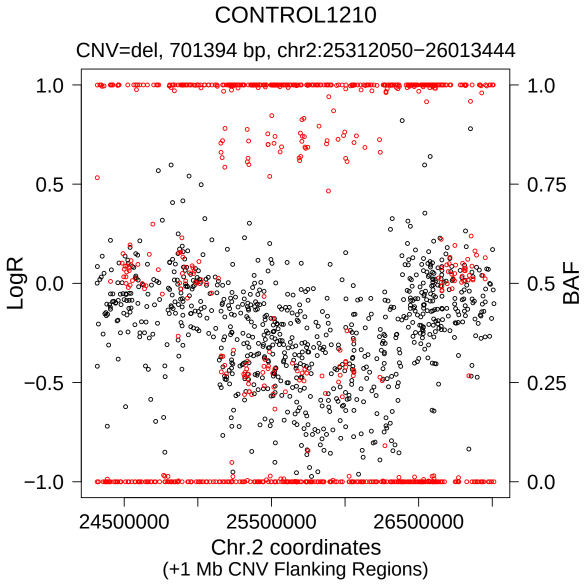

Figure 9: New mosaic rearrangement found after re-analyzing SNP arrary data described elsewhere (Rodriguez-Santiago et al. 2010). The plot shows a chromosome 2 deletion in sample CONTROL1210 by using MAD. This was not detected using ad-hoc tools. Red dots represent B-allele frequency (BAF), while black dots show log2ratio (logR) values.

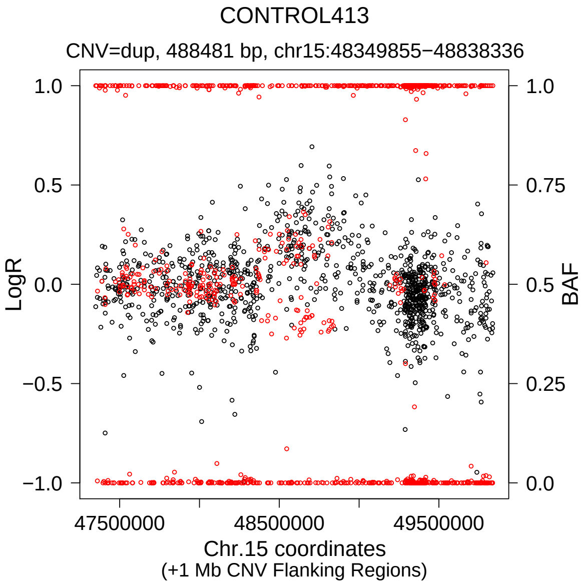

Figure 10: New mosaic rearrangement found after re-analyzing SNP array data described elsewhere (Rodriguez-Santiago et al. 2010). The plot shows a chromosome 15 duplication in sample CONTROL413 by using MAD. This was not detected using ad-hoc tools. Red dots represent B-allele frequency (BAF), while black dots show log2ratio (logR) values.

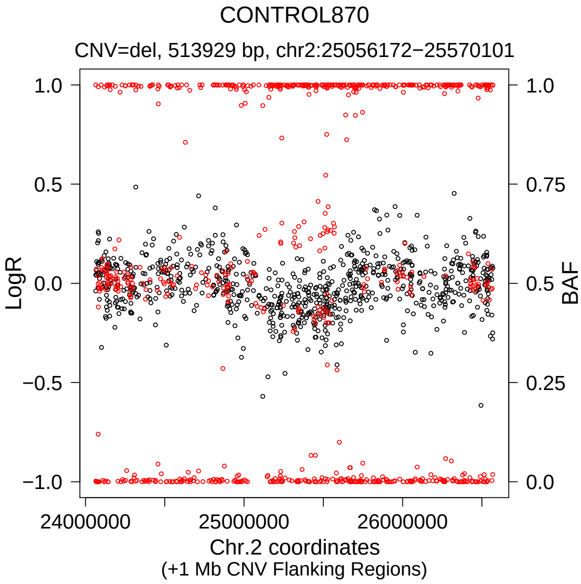

Figure 11: New mosaic rearrangement found after re-analyzing SNP array data described elsewhere (Rodriguez-Santiago et al. 2010). The plot shows a chromosome 2 deletion in sample CONTROL870 by using MAD. This was not detected using ad-hoc tools. Red dots represent B-allele frequency (BAF), while black dots show log2ratio (logR) values.

5 Validation of new mosaic abnormalities detected using MAD with MLPA experiments

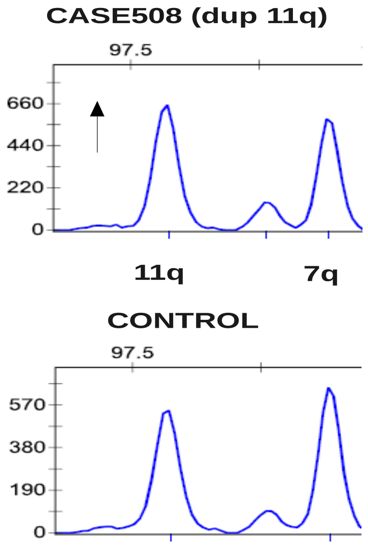

Figure 12: MLPA result of sample CASE508. MLPA abnormal peaks confirmed either a gain of genetic material consistent with a mosaic regional CNV in the individuals shown with respect to the control sample.

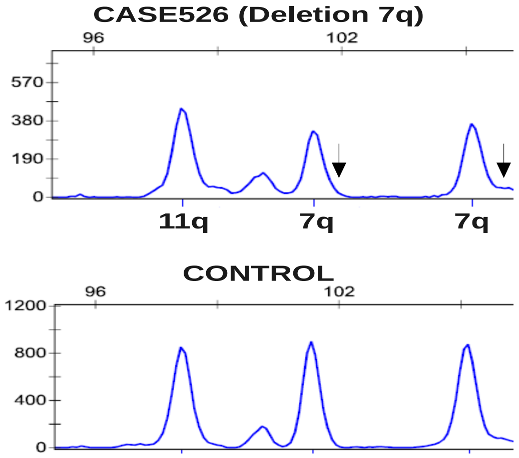

Figure 13: MLPA result of sample CASE526. MLPA abnormal peaks confirmed either a loss of genetic material consistent with a mosaic regional CNV in the individuals shown with respect to the control sample.

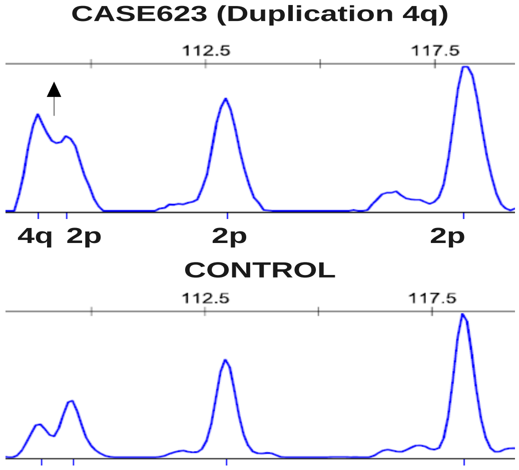

Figure 14: MLPA result of sample CASE623. MLPA abnormal peaks confirmed either a gain of genetic material consistent with a mosaic regional CNV in the individuals shown with respect to the control sample.

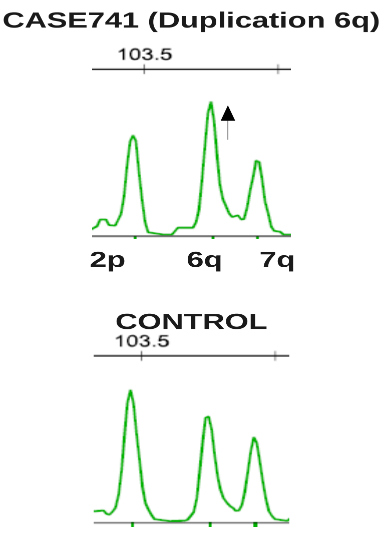

Figure 15: MLPA result of sample CASE741. MLPA abnormal peaks confirmed either a gain of genetic material consistent with a mosaic regional CNV in the individuals shown with respect to the control sample.

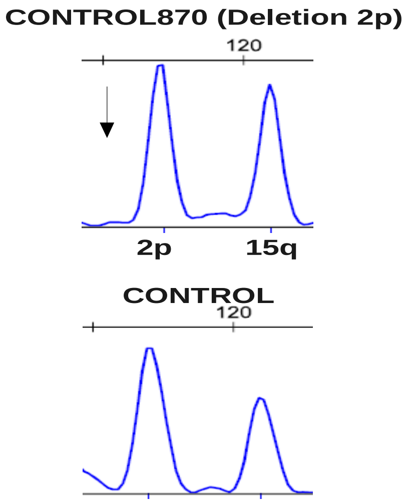

Figure 16: MLPA result of sample CONTROL870. MLPA abnormal peaks confirmed either a loss of genetic material consistent with a mosaic regional CNV in the individuals shown with respect to the control sample.

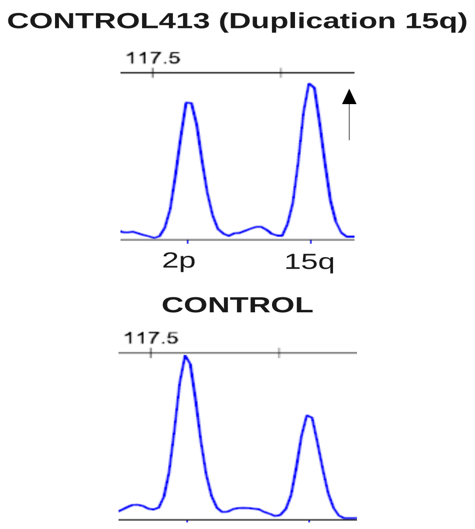

Figure 17: MLPA result of sample CONTROL413. MLPA abnormal peaks confirmed either a gain of genetic material consistent with a mosaic regional CNV in the individuals shown with respect to the control sample.
