# Supplementary material for: A fast and accurate method to detect allelic genomic imbalances underlying mosaic rearrangements using SNP array data
Source: BMC Bioinformatics. 2011 May 17;12:166. doi: 10.1186/1471-2105-12-166 (PMC3118168; doi:10.1186/1471-2105-12-166)
Supplement: Additional file 2 — Recommended parameters for a preliminary scan using different Illumina platforms. [file 1471-2105-12-166-S2.PDF]

Platform	a	T	MinSegLen
317k	0.8	9	300
370CNV	0.8	9	300
550k	0.8	8	300
1M	0.8	7.5	500

Table S1: Recommended parameters fo a preliminary scan using different Illumina platforms
