# Supplementary material for: A fast and accurate method to detect allelic genomic imbalances underlying mosaic rearrangements using SNP array data
Source: BMC Bioinformatics. 2011 May 17;12:166. doi: 10.1186/1471-2105-12-166 (PMC3118168; doi:10.1186/1471-2105-12-166)
Supplement: Additional file 3 — Comparison between MAD findings and chromosomal abnormalities previously described in HapMap individuals also analysed in Redon et al., Nature, 2006. [file 1471-2105-12-166-S3.PDF]

Supplementary Table 1. Comparison between MAD findings and chromosomal abnormalities previously described in HapMap individuals also analysed in Redon *et al*. Supplementary Figures contain the plots showing LRR and BAF values for these regions. Discrepancies between MAD results and karyotype may be due tousing different source of DNA material.

Sample	CNVs in Redon *et al* [1]	Kariotype Abnormalities in Redon *et al* [1]	Result (MAD)
NA07345	del(4)(q32.1q32.1)	del(9)(p24.1p24.1); 46,XX	No calls. Reason: not mosaic deletions (absence of het. Probes)
NA07348	del(4)(q35.2q35.2)	del(X); 45,X,-X[4]/46,XX[2]	No calls. Not mosaic (absence of het. Probes)
NA10859	del(8)(p23.2)	del(X); 46,XX	No calls. Not mosaic (absence of het. Probes)
NA11882	dup(19)(q13.41q13.43)	46,XX	Mosaic duplication 19q
NA12056	dup(16)(q23.2q24.3)	46,XY	No calls, normal plots
NA12057	del(1)(q44)	47,XX,+12[10]/46,XX[1]	No calls, normal plots
NA12248	dup(9),dup(12),dup(14),dup(22)	50,XY,+9,+12,+14,+22[1]/50,sl,add(8)(q24)[3]/	Trisomies chr. 9 and 14 called. Chr. 12 and 22 by plotting whole chromosome (low %cells affected)
NA12875	dup(2)	47,XX,+2[6]/46,XX[1]	Mosaic trisomy chr. 2
NA12891	del(15)(q25.2q25.2),del(19)(q13.12q13.12)	46,XY	No calls. Reason: not mosaic deletions (absence of het. Probes)
NA18507	del(7)(p11.1p11.1)	dup(X); 46,XY	No calls, normal plots
NA18529	del(16)(q11.2q24.3)	46,XX,inv(6)(p11q27)[7]/46,sl,-16,+mar[3]	No calls, normal plots
NA18852	del(3)(q24q24), del(13)(q32.2q32.2)	46,XX	No calls. Not mosaic (absence of het. Probes)
NA18855	del(18)(q22.1q22.1)	47~51,XX,+X[2],+5[4],+9[1],+11[9],+12[5],+17[3][cp9];	
NA18956	dup(4)(q31.3q35.2)	46,XX	No calls, normal plots
NA18987	del(12)	46,XX	No calls, normal plots
NA19140	dup(9)	46,XX	No calls, normal plots
NA19193	dup(12)	47,XX,+12	Mosaic trisomy chr. 12

Chr: chromosome, del: deletion, dup: duplication

1: Redon R, *et al*. Global variation in copy number in the human genome. Nature. 2006 Nov 23;444(7118):444-54.

2: Mosaic event different, not detected in Redon *et al*.
