# Supplementary material for: A fast and accurate method to detect allelic genomic imbalances underlying mosaic rearrangements using SNP array data
Source: BMC Bioinformatics. 2011 May 17;12:166. doi: 10.1186/1471-2105-12-166 (PMC3118168; doi:10.1186/1471-2105-12-166)
Supplement: Additional file 4 — Additional analysis using PennCNV to discard mosaic detected with MAD with consitutional duplications. [file 1471-2105-12-166-S4.PDF]

Supplementary Table 2. Many of the mosaic duplications called by applying MAD software to samples used elsewhere[1] might be not mosaic but consitutional duplications. All of them are called by HMM methods such as PennCNV[2] but a significant proportion (29 out of 98) had been discarded by using stringent filtering procedures[3].

		MAD						%cell	Overlap	Overlap	PennCNV[2]			Overlap	After
Sample	Chr	Start	Size	Probes	qqBdev	LRR	Bdev	estimation	bp MAD-SD	% MAD-SD	Start	Size	Probes	MAD-PennCNV	filtering[3]
CASE2	1	17,174,259	222,338	110	0.78	0.12	0.12	60%	0	0%	16,919,026	528,717	181	100%	Filtered
CASE174	2	8,674	352,474	170	0.33	0.19	0.06	29%	36,318	10%	150,894	213,193	86	60%	Filtered
CONTROL173	6	1,380,219	253,171	163	0.32	0.17	0.08	38%	2,333	1%	1,123,561	531,142	314	100%	
CONTROL408	6	57,688,739	4,393,563	228	0.31	0.10	0.07	33%	948,850	22%	61,942,912	1,017,885	225	23%	centromere
CONTROL171	6	57,747,544	4,279,948	203	0.23	0.13	0.06	28%	948,850	22%	62,020,891	939,906	215	22%	centromere
CONTROL1094	6	168,078,038	241,638	179	0.50	0.17	0.11	54%	0	0%	168,076,803	243,507	181	100%	
CONTROL56	6	168,078,038	241,638	179	0.76	0.18	0.12	65%	0	0%	168,075,787	264,615	198	100%	
CONTROL1152	6	168,122,631	217,460	161	0.37	0.16	0.07	34%	0	0%	168,076,803	263,599	197	100%	Filtered
CASE229	6	168,137,772	202,424	141	0.59	0.17	0.10	50%	0	0%	168,079,657	260,745	192	100%	
CONTROL1158	6	168,138,806	201,285	139	0.73	0.19	0.11	58%	0	0%	168,075,787	264,305	196	100%	Filtered
CONTROL1162	6	168,138,806	180,870	124	0.74	0.18	0.12	61%	0	0%	168,075,787	264,615	198	100%	Filtered
CONTROL721	9	33,496,670	519,868	153	0.42	0.16	0.08	38%	284,814	55%	33,647,318	646,605	185	100%	
CASE261	9	125,796,461	266,880	124	0.47	0.13	0.10	49%	0	0%	125,617,098	458,485	184	100%	
CASE1107	10	135,074,950	297,794	161	0.37	0.01	0.06	29%	121,799	41%	135,092,863	137,627	116	46%	Filtered
CONTROL111	10	135,074,950	297,794	161	0.31	0.17	0.05	22%	121,799	41%	135,092,863	137,627	116	46%	
CONTROL65	10	135,075,656	297,088	160	0.36	0.14	0.06	26%	121,799	41%	135,092,863	142,910	117	48%	
CONTROL388	10	135,077,234	295,510	158	0.29	0.15	0.05	23%	121,799	41%	135,092,863	137,627	116	47%	
CASE985	10	135,084,570	288,174	155	0.31	0.17	0.06	28%	121,799	42%	135,092,863	137,627	116	48%	
CONTROL739	10	135,084,570	288,174	155	0.29	0.08	0.05	22%	121,799	42%	135,092,863	137,627	116	48%	
CASE56	10	135,085,880	286,864	154	0.76	0.17	0.10	50%	121,799	42%	135,092,863	137,627	116	48%	
CONTROL227	10	135,085,880	286,864	154	0.36	0.14	0.06	26%	121,799	42%	135,092,863	137,627	116	48%	
CASE395	10	135,092,863	279,881	153	0.27	0.11	0.05	21%	115,077	41%	135,092,863	137,627	116	48%	Filtered
CASE659	10	135,092,863	279,881	153	0.42	0.16	0.06	29%	115,077	41%	135,092,863	142,910	117	51%	Filtered
CONTROL737	10	135,092,863	279,881	153	0.29	0.11	0.05	24%	115,077	41%	135,092,863	129,231	111	48%	Filtered
CONTROL12	10	135,092,863	279,881	153	0.27	0.17	0.05	22%	115,077	41%	135,092,863	131,499	116	47%	Filtered
CASE1080	10	135,092,863	279,881	153	0.36	0.19	0.06	25%	115,077	41%	135,092,863	137,627	116	49%	

CASE1081	10	135,092,863	279,881	153	0.52	0.19	0.09	44%	115,077	41%	135,092,863	137,627	116	49%	
CASE1122	10	135,092,863	279,881	153	0.31	0.17	0.05	22%	115,077	41%	135,092,863	142,910	117	51%	
CASE1130	10	135,092,863	279,881	153	0.29	0.18	0.05	24%	115,077	41%	135,092,863	137,627	116	49%	
CASE177	10	135,092,863	279,881	153	0.30	0.18	0.05	22%	115,077	41%	135,092,863	137,627	116	49%	
CASE335	10	135,092,863	279,881	153	0.30	0.19	0.05	22%	115,077	41%	135,092,863	137,627	116	49%	
CASE440	10	135,092,863	279,881	153	0.30	0.16	0.05	24%	115,077	41%	135,092,863	137,627	116	49%	
CASE722	10	135,092,863	279,881	153	0.31	0.10	0.05	24%	115,077	41%	135,092,863	137,627	116	49%	
CASE760	10	135,092,863	279,881	153	0.33	0.17	0.06	25%	115,077	41%	135,092,863	137,627	116	49%	
CASE927	10	135,092,863	279,881	153	0.56	0.18	0.09	46%	115,077	41%	135,092,863	137,627	116	49%	
CONTROL159	10	135,092,863	279,881	153	0.32	0.19	0.05	24%	115,077	41%	135,092,863	150,343	119	54%	
CONTROL21	10	135,092,863	279,881	153	0.32	0.15	0.06	25%	115,077	41%	135,092,863	142,910	117	51%	
CONTROL235	10	135,092,863	279,881	153	0.36	0.14	0.06	27%	115,077	41%	135,092,863	137,627	116	49%	
CONTROL241	10	135,092,863	279,881	153	0.28	0.17	0.06	25%	115,077	41%	135,092,863	137,627	116	49%	
CONTROL401	10	135,092,863	279,881	153	0.30	0.14	0.05	23%	115,077	41%	135,092,863	137,627	116	49%	
CONTROL492	10	135,092,863	279,881	153	0.33	0.18	0.05	23%	115,077	41%	135,092,863	137,627	116	49%	
CONTROL500	10	135,092,863	279,881	153	0.30	0.16	0.05	22%	115,077	41%	135,092,863	137,627	116	49%	
CONTROL52	10	135,092,863	279,881	153	0.26	0.09	0.06	27%	115,077	41%	135,092,863	137,627	116	49%	
CONTROL562	10	135,092,863	279,881	153	0.55	0.14	0.10	48%	115,077	41%	135,092,863	137,627	116	49%	
CONTROL754	10	135,092,863	279,881	153	0.30	0.14	0.05	22%	115,077	41%	135,092,863	137,627	116	49%	
CONTROL971	10	135,092,863	279,881	153	0.35	0.19	0.05	23%	115,077	41%	135,092,863	137,627	116	49%	
CASE844	10	135,100,199	272,545	151	0.42	0.19	0.06	28%	108,017	40%	135,092,863	137,627	116	49%	Filtered
CONTROL1209	10	135,100,199	272,545	151	0.34	0.19	0.06	25%	108,017	40%	135,092,863	137,627	116	49%	Filtered
CONTROL556	10	135,100,199	272,545	151	0.36	0.15	0.07	30%	108,017	40%	135,092,863	126,660	97	46%	Filtered
CONTROL942	10	135,100,199	272,545	151	0.44	0.15	0.08	37%	108,017	40%	135,092,863	137,627	116	50%	
CONTROL506	10	135,106,317	266,427	149	0.28	0.17	0.05	20%	108,017	41%	135,092,863	137,627	116	46%	Filtered
CONTROL138	10	135,106,317	266,427	149	0.28	0.19	0.06	25%	108,017	41%	135,092,863	137,627	116	52%	
CONTROL788	10	135,106,752	265,992	148	0.26	0.14	0.05	22%	108,017	41%	135,092,863	137,627	116	52%	
CASE672	10	135,109,510	263,234	145	0.34	0.19	0.06	25%	108,017	41%	135,095,834	134,656	115	51%	
CASE1013	10	135,129,800	242,944	136	0.32	0.14	0.06	26%	108,017	44%	135,100,199	135,574	115	56%	
CONTROL1	11	133,853,184	592,442	134	0.33	0.11	0.06	25%	65,507	11%	133,853,184	305,785	172	100%	Filtered
CASE387	11	133,853,184	592,442	300	0.36	0.14	0.08	35%	65,507	11%	133,853,184	373,879	209	63%	
CONTROL187	11	133,853,501	592,125	299	0.40	0.17	0.07	30%	65,507	11%	133,853,184	373,879	209	63%	
CASE1043	11	133,853,636	591,990	298	0.46	0.19	0.07	32%	65,507	11%	133,853,501	373,181	207	63%	

CASE173	11	133,853,636	591,990	298	0.30	0.18	0.06	25%	65,507	11%	133,853,184	370,244	206	63%	
CONTROL228	11	133,853,636	343,754	190	0.70	0.17	0.10	51%	0	0%	133,853,636	349,951	195	100%	
CONTROL685	11	133,853,636	591,990	298	0.44	0.16	0.07	33%	65,507	11%	133,853,501	370,779	206	63%	
CONTROL578	11	133,853,860	591,766	297	0.45	0.15	0.08	38%	65,507	11%	133,853,184	371,096	207	63%	Filtered
CONTROL773	11	133,853,860	591,766	297	0.38	0.07	0.07	31%	65,507	11%	133,853,636	373,427	207	63%	
CONTROL1042	11	133,874,627	570,999	286	0.46	0.13	0.08	36%	65,507	11%	133,853,184	373,879	209	65%	Filtered
CONTROL218	11	133,874,627	570,999	286	0.45	0.14	0.07	33%	65,507	11%	133,853,184	383,856	211	67%	
CASE808	11	133,879,982	565,644	283	0.48	0.09	0.07	34%	65,507	12%	133,853,184	373,879	209	66%	
CASE55	11	133,914,862	275,262	158	0.60	0.19	0.08	40%	0	0%	133,889,790	459,231	242	100%	
CONTROL735	11	133,986,613	459,013	234	0.51	0.19	0.08	39%	65,507	14%	133,853,184	373,879	209	81%	
CASE1109	12	21,054	658,431	305	0.18	0.13	0.04	18%	32,438	5%	139,792	547,509	251	53%	
CASE833	14	42,901,587	415,904	100	0.66	0.07	0.13	73%	0	0%	42,897,456	420,036	101	100%	
CASE1078	15	18,275,409	2,231,045	303	0.33	0.13	0.08	38%	1,764,610	79%	20,305,097	513,009	191	23%	
CONTROL645	15	18,275,409	2,281,234	332	0.22	0.12	0.06	26%	1,764,610	77%	20,303,106	497,459	190	22%	
CASE1202	15	20,335,459	275,967	149	0.72	0.16	0.14	74%	29,653	11%	20,203,694	664,536	206	100%	Filtered
CASE806	15	99,863,811	459,820	152	0.30	0.13	0.06	27%	168,176	37%	99,862,888	171,379	88	37%	Filtered
CONTROL907	16	15,036,960	1,149,824	655	0.37	0.18	0.09	42%	352,938	31%	15,369,798	818,858	598	71%	
CONTROL626	16	15,446,828	534,329	280	0.51	0.16	0.09	45%	0	0%	15,387,380	837,759	614	100%	
CONTROL626	16	15,984,272	205,536	297	0.24	0.12	0.05	23%	0	0%	15,387,380	837,759	614	100%	
CASE756	17	28,984,361	975,765	505	0.45	0.13	0.09	46%	19,395	2%	28,984,361	814,527	443	83%	Filtered
CASE202	22	14,430,353	1,238,635	143	0.29	0.11	0.07	34%	1,039,439	84%	15,437,138	237,114	78	19%	Filtered
CASE319	22	14,430,353	1,209,601	125	0.34	0.13	0.08	38%	1,039,439	86%	14,802,908	837,047	96	69%	Filtered
CASE843	22	14,430,353	1,243,898	145	0.39	0.18	0.08	35%	1,039,439	84%	15,434,027	240,225	81	19%	Filtered
CASE922	22	14,430,353	1,235,976	142	0.24	0.18	0.06	26%	1,039,439	84%	15,284,080	263,929	85	21%	Filtered
CONTROL1147	22	14,430,353	1,250,864	146	0.25	0.16	0.06	27%	1,039,439	83%	15,235,618	438,634	103	35%	Filtered
CONTROL17	22	14,430,353	1,235,596	141	0.32	0.16	0.07	33%	1,039,439	84%	15,284,080	390,172	99	32%	Filtered
CONTROL560	22	14,430,353	1,243,898	145	0.35	0.09	0.09	43%	1,039,439	84%	23,980,406	256,398	94	21%	Filtered
CONTROL992	22	14,430,353	1,235,596	141	0.30	0.18	0.06	28%	1,039,439	84%	15,284,080	319,752	92	26%	Filtered
CASE15	22	14,430,353	1,235,976	142	0.28	0.15	0.07	30%	1,039,439	84%	15,634,399	39,853	24	3%	
CASE379	22	14,430,353	1,238,635	143	0.26	0.16	0.07	31%	1,039,439	84%	15,437,138	237,114	78	19%	
CASE585	22	14,430,353	1,235,976	142	0.27	0.17	0.07	31%	1,039,439	84%	15,634,399	39,853	24	3%	
CASE873	22	14,430,353	1,235,976	142	0.37	0.17	0.07	34%	1,039,439	84%	15,435,569	238,683	79	19%	
CONTROL1010	22	21,592,360	388,627	170	0.88	0.19	0.15	87%	1,874	0%	21,441,199	538,044	206	100%	Filtered

CASE1048	22	23,413,914	1,374,474	701	0.44	0.16	0.08	40%	284,114	21%	41,123,507	123,592	48	9%	
CONTROL1063	22	23,948,890	290,314	110	0.49	0.19	0.08	38%	209,131	72%	23,991,725	252,869	102	87%	
CASE2	22	23,983,992	256,675	102	0.75	0.12	0.11	56%	184,548	72%	23,983,992	260,602	103	100%	Filtered
CASE1048	22	24,793,024	344,093	203	0.69	0.17	0.12	64%	0	0%	23,413,914	2,449,013	1370	100%	
CASE1048	22	25,140,315	183,391	116	0.33	0.11	0.07	33%	1,196	1%	23,413,914	2,449,013	1370	100%	
CASE1048	22	25,327,482	511,376	333	0.66	0.14	0.11	57%	0	0%	23,413,914	2,449,013	1370	100%	

bp: base pairs, SD: Segmental Duplication

% cell estimation was calculated as described elsewhere[1]

"Overlap bp MAD-SD" and "Overlap % MAD-SD" columns show the overlap between MAD segments and genomic segmental duplications.

"Overlap MAD-PennCNV" column shows the percentage of the called MAD segment present in a CNV call obtained when using PennCNV[2].

1. Rodríguez-Santiago B, *et al*. Mosaic uniparental disomies and aneuploidies as large structural variants of the human genome. Am J Hum Genet. 2010 Jul 9;87(1):129-38.

2. Wang K, *et al*. PennCNV: an integrated hidden Markov model designed for high-resolution copy number variation detection in whole-genome SNP genotyping data. Genome Research. 2007. 17:1665-1674.

3. Itsara A, *et al*. Population analysis of large copy number variants and hotspots of human genetic disease. Am J Hum Genet. 2009 Feb;84(2):148-61.
