# Supplementary material for: A fast and accurate method to detect allelic genomic imbalances underlying mosaic rearrangements using SNP array data
Source: BMC Bioinformatics. 2011 May 17;12:166. doi: 10.1186/1471-2105-12-166 (PMC3118168; doi:10.1186/1471-2105-12-166)
Supplement: Additional file 5 — User's guide of an R package that implements MAD algorithm including some real data examples. [file 1471-2105-12-166-S5.PDF]

User's Guide: A fast R program to detect allelic imbalances using Illumina data and GADA algorithm

Juan R González, Benja Rodríguez-Santiago, Alejandro Cáceres, Roger Pique-Regi, Nathaniel Rothman, Stephen J Chanock, Lluís Armengol, Luis Pérez-Jurado

Center for Research in Environmental Epidemiology (CREAL)
http://www.creal.cat/jrgonzalez/software.htm

September 10, 2010

1 Introduction

We have developed an algorithm to detect allelic imbalances using Illumina data. In general words, the method detects segments were B-deviation is different from 0. Segmentation procedure is carried out using GADA algorithm (R package available at http://groups.google.com/group/gadaproject (Pique-Regi R, Caceres A, Gonzalez JR. BMC Bioinformatics, 2010). The functions to detect allelic imbalances (described in this manual) are available in a new version of the package (it can be download from http://www.creal.cat/jrgonzalez/GADA/gada_0.9-4.tar.gz). Herein, we briefly describe how to use the main functions by using a real data set of 5 samples (CASE369.txt, CASE371.txt, CASE377.txt, CONTROL152.txt, CONTROL191.txt) that can be downloaded from:
http://www.creal.cat/jrgonzalez/GADA/rawData_example_MAD.zip

2 Getting Started

The package can be installed by executing

```
> install.packages("gada_0.9-4.tar.gz", repos=NULL)
```

Then, it can be loaded by typing

```
> library(gada)
```

3 Raw data format

The package enforces a strict directory structure on the working directory to perform the analysis of multiple samples. However, the only required directory to be set up by the user is that containing the raw data. This is an example of how data are organized before the analysis is completed

```
|-- rawData
|   |-- CASE369.txt
|   |-- CASE371.txt
|   |-- CASE377.txt
```

```
|   |-- CONTROL152.txt
|   |-- CONTROL191.txt
```

Notice that `rawData` directory contains all data files corresponding to each individual from a particular assay. Each text file can be prepared by using the BeadStudio tool, which is available at `http://www.illumina.com/`, and must have the following structure (this corresponds to the file `CASE369.txt`):

```
Name      Chr       Position        Log.R.Ratio     B.Allele.Freq   GType
rs758676            7       12878632         0.1401  0.4977  AB
rs3916934           13      103143536        0.3934  0.461   AA
rs2711935           4       38838852        -0.1091  0.0026  AA
rs17126880          1       64922104         0.0478  0.991   AA
rs12831433          12      4995220         -0.1661  0       AA
rs12797197          11      81359825         0.1216  0       AB
rs11002902          10      80702561        -0.1385  0.4818  AA
rs3924674           5       76476752         0.0485  0.9853  AB
rs2147587           1       95487472         0.1156  4e-04   AA
```

GADA manual (available at `http://groups.google.com/group/gadaproject/web/gada_manual.pdf`) describes how to import Illumina data using BeadStudio tool (Section 4.1). Data files can be either tab- or with space-delimited files. The `R` functions consider tab-delimiter option but it can be easily changed. It is important to mention that all files included in the folder `rawData` must belong to the same type of array (e.g. same number of probes) because the annotation data is obtained from one of these files. If samples are going to be analyzed using different platforms, the best option is to create another working directory containing another `rawData` folder.

4 Importing a collection of Illumina array data

Data can be imported to `R` as following:

```
example<-setupParGADA.B.deviation(NumCols=6, GenoCol=6, BAFcol=5, log2ratioCol=4)

Creating object with annotation data ...
Read 3218460 items
Creating object with annotation data ...done

Creating objects of class setupGADA for all input files...
  Applying setupGADA.B.deviation for 5 samples ...
  Importing array:  CASE369.txt ... Read 6436920 items
   Array # 1 ...done
  Importing array:  CASE371.txt ... Read 6436920 items
   Array # 2 ...done
  Importing array:  CASE377.txt ... Read 6436920 items
   Array # 3 ...done
  Importing array:  CONTROL152.txt ... Read 6436920 items
   Array # 4 ...done
  Importing array:  CONTROL191.txt ... Read 6436920 items
   Array # 5 ...done
  Applying setupGADA.B.deviation for 5 samples ... done
Creating objects of class setupGADA for all input files... done
```

This function requires the following information: `NumCols` gives the number of columns each file has, `GenoCol` indicates which colum contains the information about genotypes, `BAFcol` denotes the file containing the B-allele frequency, and `log2ratioCol` informs which column contains the $\log_2$ ratio instensities.

The object `example` must be created because is required for further analysis. It contains the following information:

```
>  example
[1] "/home/jrgonzalez/CREAL/CRG/lluis/Mosaicismo/multi"
attr(,"class")
[1] "parGADA"
attr(,"type")
[1] "Illumina"
attr(,"labels.samples")
[1] "CASE369"    "CASE371"    "CASE377"    "CONTROL152" "CONTROL191"
attr(,"Samples")
[1] 5
attr(,"b.deviation")
[1] TRUE
```

5 Segmentation procedure

Segmentation is made of two consecutive algorithms implemented in two separate R-functions within the `gada` package. The first function (`parSBL`) uses sparse Bayesian learning (SBL) to discover the most likely positions and magnitudes for a segment, i.e. the breakpoints. The SBL model is governed by a hierarchical Bayesian prior, which is uninformative with respect to the location and magnitude of the copy number changes but restricts the total number of breakpoints. Sensitivity, given by the maximum breakpoint sparseness, is controlled by the hyperparameter `aAlpha`. The second function (`parBE`) is an algorithm that uses a backward elimination (BE) strategy to rank the statistical significance of each breakpoint obtained from `SBL`. We have programmed another function called `parBE.B.deviation` to detect segments that can be considered as an allelic imbalance. The results from `parSBL` and `parBE.B.deviation` are stored in separate files, one for each sample, in a folder called `SBL` .

Therefore, the segmentation procedure can be performed by executing

```
> parSBL(example, estim.sigma2=TRUE, aAlpha=0.8)
Creating SBL directory ...done
Retrieving annotation data ...done
Segmentation procedure for 5 samples ...
   Array # 1 ...    The estimated sigma2 = 0.8116668
   Array # 1 ...done
   Array # 2 ...    The estimated sigma2 = 0.7624304
   Array # 2 ...done
   Array # 3 ...    The estimated sigma2 = 0.724603
   Array # 3 ...done
   Array # 4 ...    The estimated sigma2 = 0.6284825
   Array # 4 ...done
   Array # 5 ...    The estimated sigma2 = 0.3593857
   Array # 5 ...done
Segmentation procedure for 5 samples ...done
```

and

```
> parBE.B.deviation(example, T=9, MinSegLen=75)
```

```
Retrieving annotation data ...done
Backward elimination procedure for 5 samples ...
   Array # 1 ... ---------------------------------------
Sparse Bayesian Learnig (SBL) algorithm
Backward Elimination procedure with T=9 and minimun length size=75
 Number of segments =  35
 Base Amplitude of copy number 2: chr 1:22:0.2598, X=NA, Y=NA
   Array # 2 ... ---------------------------------------
Sparse Bayesian Learnig (SBL) algorithm
Backward Elimination procedure with T=9 and minimun length size=75
 Number of segments =  34
 Base Amplitude of copy number 2: chr 1:22:0.2355, X=NA, Y=NA
   Array # 3 ... ---------------------------------------
Sparse Bayesian Learnig (SBL) algorithm
Backward Elimination procedure with T=9 and minimun length size=75
 Number of segments =  28
 Base Amplitude of copy number 2: chr 1:22:0.2077, X=NA, Y=NA
   Array # 4 ... ---------------------------------------
Sparse Bayesian Learnig (SBL) algorithm
Backward Elimination procedure with T=9 and minimun length size=75
 Number of segments =  36
 Base Amplitude of copy number 2: chr 1:22:0.1624, X=NA, Y=NA
   Array # 5 ... ---------------------------------------
Sparse Bayesian Learnig (SBL) algorithm
Backward Elimination procedure with T=9 and minimun length size=75
 Number of segments =  31
 Base Amplitude of copy number 2: chr 1:22:0.0749, X=NA, Y=NA
Backward elimination procedure for 5 samples ...done
```

As previously mentioned, the parameter `aAlpha` controls the number of breakpoints. We consider that `aAlpha=0.8` is a good choice for Illumina 1M. The parameter `T` controls the False Discovery Rate (FDR). We are currently working on estimating the FDR but the recommended settings of `aAlpha` and `T` depending on the desired level of sensitivity and FDR for a Illumina 650K data are.

(higher sensitivity , higher FDR)	$< -- >$	$(a_\alpha = 0.2, T > 3)$
	$< -- >$	$(a_\alpha = 0.5, T > 4)$
(lower sensitivity , lower FDR)	$< -- >$	$(a_\alpha = 0.8, T > 5)$

Anyway the backward elimination procedure is very fast and the user can change the parameter T and obtain different results. The argument `MinSegLen` indicates the number of consecutive probes that have a B-deviation different from 0.

6 Obtaining allelic imbalance regions

The user can type

```
> exportSegments2File(example, file="example_T_9.txt")
```

to get a file including the following information

IniProbe	EndProbe	LenProbe	qqBdev	chr	LRR	(s.e.)	Bdev	%HetWT	%Hom	State	sample
49728792	52510626	1329	-0.23	3	0.02	0.2	0.044	4.5	91.6	5	CASE369
31578652	32298368	1522	-0.24	6	0	0.14	0.043	3.8	91.6	5	CASE369
40160724	43767684	1599	-0.25	15	0.02	0.19	0.043	4.9	90.6	5	CASE369
31726669	47051222	1161	-0.28	16	0.03	0.19	0.036	3.7	88.6	5	CASE369
41256855	78653169	14847	0.17	17	0.02	0.22	0.105	0.2	73	1	CASE369
126632473	127572870	427	-0.31	7	-0.01	0.45	0.041	3.5	90.4	5	CASE371
65615750	67006562	503	-0.35	14	0.01	0.17	0.028	0.2	98.2	5	CASE371
31049772	47452926	1521	-0.27	16	-0.01	0.3	0.038	2.6	88	5	CASE371
98214394	100585331	1190	-0.15	7	-0.02	0.36	0.046	0.4	97.5	5	CONTROL152
99490091	101235631	721	-0.29	8	-0.06	0.25	0.019	3.1	93.6	1	CONTROL152
36587	38762575	17179	0.32	9	0.1	0.23	0.122	0.5	70.7	3	CONTROL152
38763251	70374589	611	-0.17	9	0.08	0.36	0.033	1.8	82.2	1	CONTROL152
70379173	140225046	27853	0.18	9	0.1	0.22	0.087	0.6	71.1	3	CONTROL152
31688557	46990324	1151	-0.23	16	-0.05	0.29	0.022	2.2	91.3	1	CONTROL152
44638889	48596642	1763	0.27	20	-0.22	0.29	0.069	0.4	71.6	2	CONTROL191

The column `State` is a preliminary attempt to classify mosaic alterations after MAD calling process based on log2-ratio segment values (LRR) together with the percentage of normal heterozygous (BAF ~ 0.5) and homozygous probes. The number codes correspond to the following abnormalities: UPD (1), deletion (2), duplication (3), trisomy (4) and LOH (5). It is recommendable to check called segments by making chromosome plots and observing LRR and BAF values to confirm those segments with limit cut-off values.

Other regions are found if one changes the `T` parameter

```
> parBE.B.deviation(example, T=7, MinSegLen=75)
```

and then execute

```
> exportSegments2File(example, file="example_T_7.txt")
```

Finally, if the user is interested in looking a given region with regard to B-allele frequency and `log2ratio`, the `plotChr` function can be used to get the figure 1

```
> plotChr(example, sample="CONTROL191", chr=20)
```

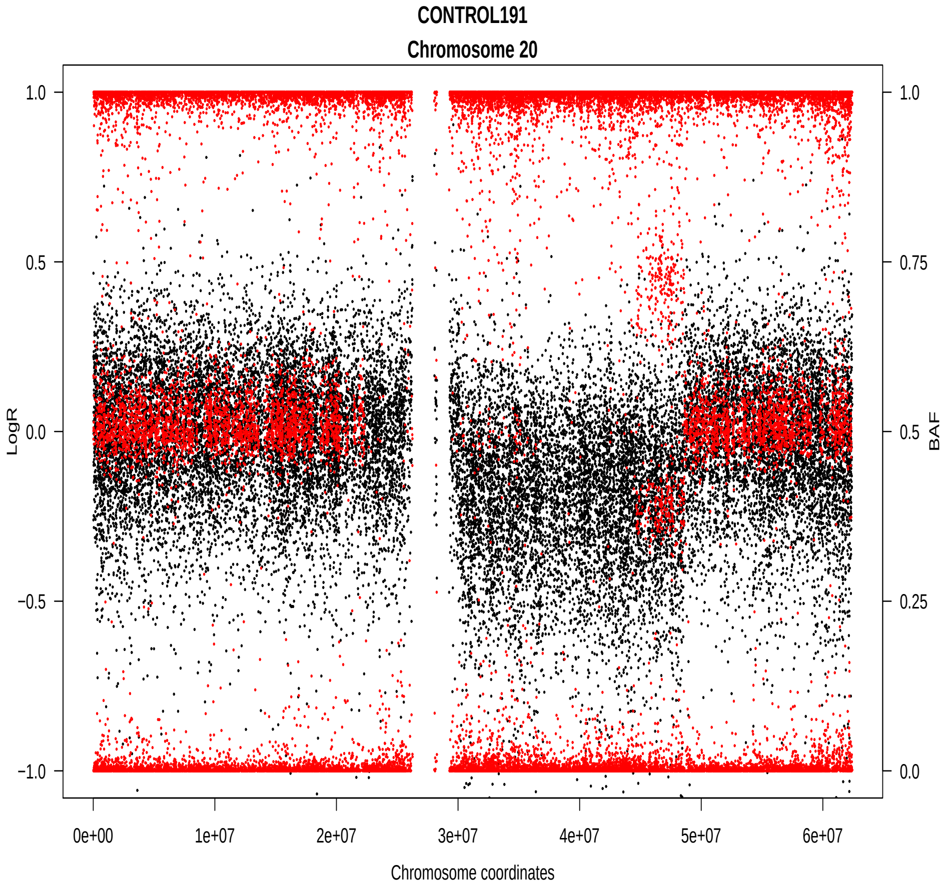

Figure 1: B-allele frequency (red points) and `log2ratio` (dark colors) for individual CONTROL191 and chromosome 20
